# Development of an Antimicrobial Nanoemulsion Based on *Cordia verbenacea* Essential Oil: Properties, Stability, Irritability, and In Vitro Skin Permeation

**DOI:** 10.3390/pharmaceutics18030313

**Published:** 2026-02-28

**Authors:** Franklyn Santos da Silva, Breno Noronha Matos, Rebeca Dias dos Santos, Venancio Alves Amaral, Marta Oliveira de Araújo, Bruno Alcântara do Prado, Cinara Medeiro Martins, Claudio Augusto Gomes da Camara, Marcilio Martins de Moraes, Diego Juscelino Santos Dias, Camila Oliveira Cardoso, Lívia Cristina Lira de Sá Barreto, Izabel Cristina Rodrigues da Silva, Daniela Castilho Orsi, Guilherme Martins Gelfuso

**Affiliations:** 1 Laboratory of Quality Control, University of Brasília (UNB/FCE), Brasília 72220-900, DF, Brazil; franklyn.silva@aluno.unb.br (F.S.d.S.); rebecadias123@gmail.com (R.D.d.S.); oa.martaaraujo@gmail.com (M.O.d.A.); prado.bruno@aluno.unb.br (B.A.d.P.); cinaramedeiros1313@gmail.com (C.M.M.); djsdias@unb.br (D.J.S.D.); belbiomedica@gmail.com (I.C.R.d.S.); 2 Laboratory of Food, Drugs, and Cosmetics, University of Brasília (UNB/FS), Brasília 72220-900, DF, Brazil; brenomatos15@hotmail.com (B.N.M.); venancio.aa@gmail.com (V.A.A.); ca_milaoc@hotmail.com (C.O.C.); liviabarreto@unb.br (L.C.L.d.S.B.); gmgelfuso@unb.br (G.M.G.); 3 Laboratory of Bioactive Natural Products, Department of Chemistry, Federal Rural University of Pernambuco, Recife 70910-900, PE, Brazil; claudio.camara@ufrpe.br (C.A.G.d.C.); marcilio.moraes@ufrpe.br (M.M.d.M.)

**Keywords:** *Varronia curassavica* essential oil, erva baleeira, antimicrobial activity, nanoemulsion, hen’s egg test on the chorioallantoic membrane, skin permeation

## Abstract

**Background/Objectives:** This study aimed to evaluate the chemical composition and antimicrobial activity of *Cordia verbenacea* essential oil (EOCV) and to develop and characterize a gel-based nanoemulsion of *C. verbenacea* essential oil (NECV). **Methods:** The EOCV was chemically characterized by gas chromatography (GC-FID and GC-MS). The nanoemulsion was prepared using EOCV, the surfactants Cremophor and Plurol Oleique, and phosphate buffer at pH 5.5, and was subjected to experiments to determine its stability, irritant potential and in vitro skin permeation. **Results:** The main chemical compounds identified in EOCV were α-pinene (33.05%) and β-caryophyllene (25.11%). The EOCV exhibited antimicrobial activity with MIC and MBC values ranging from 6.3 to 25.0 µL/mL for the yeasts *Candida albicans* and *C. krusei*, 11.3 to 25.0 µL/mL for the Gram-positive bacteria *Bacillus cereus*, *Staphylococcus aureus*, and *Enterococcus faecalis*, and 12.5 to 75.0 µL/mL for the Gram-negative bacteria *Acinetobacter baumannii*, *Klebsiella pneumoniae*, and *Escherichia coli*. The NECV showed a droplet size of 126.80 nm, a polydispersity index (PDI) of 0.49, a zeta potential of −18.50 mV and a pH of 5.3 and remained stable for 60 days of storage at 25 °C and 4 °C. The HET-CAM irritation test showed that the formulation is non-irritating. The in vitro skin permeation assay showed that the NECV penetrated the deeper layers of the skin, demonstrating its ability to overcome the *Stratum corneum* barrier. **Conclusions:** These results are highly promising regarding the potential use of NECV for topical application for the treatment of infected skin wounds.

## 1. Introduction

*Cordia verbenacea* DC, also known as *Varronia curassavica* Jacq., is an aromatic plant native to Brazil, found in various regions along the country’s coastal areas. It is popularly known as “erva baleeira” and belongs to the Boraginaceae family. The leaves of this plant are used in folk medicine in the form of extracts, decoctions, and infusions for internal or topical use in the treatment of various diseases, with antimicrobial, anthelmintic, analgesic, anti-inflammatory, and healing applications [1,2,3].

The essential oil of *C. verbenacea* (EOCV) is extracted from the leaves, and compounds such as α-pinene, β-caryophyllene, and α-humulene are representatives of the EOCV [2,3,4]. Pharmacological studies have indicated that the anti-inflammatory effects of EOCV are primarily related to two sesquiterpenes, α-humulene and β-caryophyllene [5]. and another proven pharmacological activity of EOCV is its antimicrobial action, mainly against Gram-positive bacteria and yeasts [1].

Despite the numerous biological properties of essential oils (EOs), their low solubility in water, high volatility, and environmental sensitivity limit the development of suitable pharmaceutical formulations. Thus, the production of nanoemulsions is an alternative to improve stability and ensure efficacy [6,7]. Nanoemulsions are dispersions of water-in-oil (W/O) or oil-in-water (O/W) stabilized by surfactants, whose droplets diameter of the internal phase range from 20 to 200 nm. In addition to solubilizing and stabilizing oils in aqueous dispersion, nanoemulsions increase the surface-to-volume ratio, promoting more effective cellular absorption of the actives incorporated therein [8,9,10].

Wounds have become a major challenge for healthcare systems worldwide, and many people will develop acute or chronic wounds at some point in their lives [11,12]. Microbial wound infections, a common occurrence in burn cases, represent a serious problem due to increasing antimicrobial resistance and the biofilm forming capacity of various bacteria. In recent years, the incidence of infected wounds has been steadily rising, as has the clinical and economic interest in effective therapies. Such approaches combine reduction in pathogen load in the wound with overall wound management to facilitate the healing process. The success of current therapies is challenged by the harsh conditions of the wound microenvironment, chronicity, and biofilm formation, all of which impede attainment of adequate concentrations of active antimicrobials at the site of infection. Inadequate dosing accuracy of systemically and topically applied antibiotics tends to promote the development of antimicrobial resistance, while, in the case of antiseptics, cytotoxicity remains a significant issue [13,14].

In this context, natural plant-derived substances provide safe wound-healing products and represent an excellent source for developing drugs with promising therapeutic benefits. These compounds can serve as alternative treatments to cure and manage skin disorders due to their lower side effects and favorable cost effectiveness combined with high efficacy [15]. Studies report that these compounds offer various applications and benefits, including antimicrobial activity, promotion of wound healing, treatment of burn injuries, and anti-inflammatory effects against a range of skin disorders [16]. In a recent study, the potential uses of curcumin nanoemulsion in the treatment of burn wounds were reviewed, highlighting that the nanoemulsion improved the skin permeation of curcumin, thereby enhancing its pharmacological efficacy, especially as an antimicrobial agent, making it a promising topical therapy for the treatment of infections in burn wounds [14].

Considering that no studies have been reported on nanoemulsions of *C. verbenacea* essential oil and the therapeutic properties of EOCV and the benefits of nanoemulsions for stabilizing and enhancing the delivery of oily actives to the skin, the present study developed an EOCV nanoemulsion aiming to improve its topical efficacy for potential future applications in the treatment of infected wounds. In this work, the chemical composition and antimicrobial activity of EOCV were evaluated, followed by the development of a gel-based NECV and the assessment of its stability, irritability, and vitro skin permeation.

## 2. Material and Methods

### 2.1. Identification of Major Volatile Compounds of EOCV

The EOCV was a commercial brand acquired from a company located in Santa Catarina, southern Brazil, with local production of the plants and the essential oil extracted from the leaves through steam distillation technique according to the manufacturer protocols. The EOCV was subjected to chemical characterization using gas-chromatography with flame ionization detection (GC-FID) for quantitative analysis and gas chromatography–mass spectrometry (GC-MS) for qualitative analysis. The quantitative GC analysis was carried out using a PerkinElmer Clarus 500 GC instrument (Waltham, MA, USA) equipped with a flame ionization detector (FID) and a non-polar DB-5 fused silica capillary column (30 mm × 0.25 mm × 0.25 μm) (J&W Scientific, Agilent Technologies, Santa Clara, CA, USA). The oven temperature was programmed from 60 to 240 °C at a rate 3 °C min^−1^. Injector and detector temperatures were 260 °C. Hydrogen was used as the carrier gas at a flow rate of 1 mL min^−1^ in split mode (1:30). The injection volume was 1.0 µL of diluted solution (1/100) of oil in n-hexane. The amount of each compound was calculated from GC-FID peak areas in the order of DB-5 column elution and expressed as a relative percentage of the total area of the chromatograms.

The qualitative GC-MS analysis were carried out using a CG-EM QP2010 SE Plus Shimadzu Chromatograph system (Nakagyo-ku, Kyoto, Japan) with a mass selective detector, mass spectrometer in EI 70 eV with a scan interval of 0.5 s and fragments from 40 to 550 Da. fitted with the same column and temperature program as that for the GC-FID experiments, with the following parameters: carrier gas = helium; flow rate = 1 mL min^−1^; split mode (1:30); injected in auto injector AOC-20i a volume = 1 µL of diluted solution (1/100) of oil in n-hexane.

The identification of the components was based on retention indices obtained by GC-MS, with reference to a homologous series of C8–C40 n-alkanes, calculated using the Van den Dool and Kratz equation [17], and by computer matching with the mass spectral libraries of the GC-MS data system (NIST 14 and WILEY 14th edition), as well as by co-injection with authentic standards and comparison with published mass spectra [18]. Area percentages were obtained from the GC-FID response without the use of an internal standard or correction factors. All monoterpenes and sesquiterpenes used in the identifications of volatile components were purchased from Sigma-Aldrich (São Paulo, SP, Brazil).

### 2.2. Determination of Total Phenolic Compounds and Antioxidant Activity of EOCV

The total phenolic content was determined using the Folin–Ciocalteu method [19], and the results were expressed as mg gallic acid equivalent per mL (mg GAE/mL). The antioxidant activity was evaluated using the DPPH (2,2-diphenyl-1-picrylhydrazyl) free radical scavenging assay according to Silveira et al. [20] and ABTS (2,2-azinobis-3-ethylbenzthiazoline-6-sulphonic acid) free radical scavenging assay as described by Rufino et al. [21]. The results of antioxidant activity were expressed as μmol Trolox equivalents per mL of sample (μmol TE/mL). The Folin–Ciocalteu, DPPH, and ABTS reagents were from Sigma-Aldrich (São Paulo, SP, Brazil).

### 2.3. Determination of Minimal Inhibitory Concentration (MIC) and Minimal Bactericidal Concentration (MBC) of EOCV

The microbial inocula used consisted of Gram-positive bacteria *Staphylococcus aureus* ATCC 29213, *Enterococcus faecalis* ATCC 29212 and *Bacillus cereus* ATCC 14579; Gram-negative bacteria *Acinetobacter baumannii* ATCC 19606, *Klebsiella pneumoniae* ATCC BAA 1706 and *Escherichia coli* ATCC 25922; and yeasts *Candida albicans* ATCC 10231 and *Candida krusei* ATCC 6258. The bacterial inocula were prepared by direct suspension of microbial growth in Mueller–Hinton broth (Himedia, Thane, India), and the yeasts inocula were prepared by direct suspension of microbial growth in RPMI broth (Merck, Darmstadt, Germany), with both adjusted to a turbidity equivalent to 0.5 on the McFarland scale [22,23].

The MIC and MBC of EOCV were carried out using a broth microdilution method as recommended by Clinical and Laboratories Standards Institute [22,23], with adaptations. The EOCV was diluted in different concentrations in Mueller–Hinton broth for bacteria and in RPMI broth for yeast using serial dilution (the concentrations ranged from 0.15 to 0.003 mL/mL). Then, the volume of 0.2 mL of the inoculum suspensions was distributed into a 96-well microtiter plate containing 0.2 mL of the different concentrations of EOCV. The positive control (indicating bacterial or yeast growth) consisted of 0.2 mL of inoculum and 0.2 mL of Mueller–Hinton broth (for bacteria) or 0.2 mL of RPMI broth (for yeast), while the negative control (indicating inhibition of bacterial or yeast growth) consisted of 0.2 mL of essential oil and 0.2 mL of Mueller–Hinton broth (for bacteria) or 0.2 mL of RPMI broth (for yeast). The samples were incubated for 24 h at 37 °C for bacteria and for 48 h at 37 °C for yeast.

To determine the MBC, 100 µL aliquots from each sample were spread and incubated on Potato Dextrose Agar (Himedia, Thane, India) for yeast or Mueller–Hinton Agar (Himedia, Thane, India) for bacteria and incubated for 18–24 h at 37 °C for bacteria and 48 h at 37 °C for yeast. The MBC was defined as the lowest concentration at which no visible growth was observed on the agar. For the MIC tests, 20 µL of 0.01% (*w*/*v*) resazurin dye (Sigma-Aldrich, São Paulo, SP, Brazil) was added to each well containing 100 µL of the bacterial sample, while for yeast samples, 20 µL of an aqueous 0.5% (*w*/*v*) solution of 2,3,5-triphenyl tetrazolium chloride (TTC, Merck, Darmstadt, Germany) was added to each well. Resazurin is an oxidation reduction indicator used to assess cell growth. It has a non-fluorescent blue color that becomes pink and fluorescent when reduced to resorufin by oxidoreductases within viable cells [24]. TTC is also an oxidation reduction indicator used to assess cell growth, and the reduction of tetrazolium salts from colorless aqueous solutions to intensely colored derivatives known as formazans has been the basis of its use as a redox dye for cell viability assays [25]. The MIC was defined as the lowest concentration at which there was no change in color of the redox indicators.

### 2.4. Compatibility Studies

The compatibility between EOCV and the main components selected to prepare the nanoemulsion (Cremophor and Plurol) was evaluated by thermal analysis. The components were evaluated isolated, in binary mixtures, or mixture in proportions to form the NECV. Differential scanning calorimetry (DSC) was performed using a DSC-60 instrument (Shimadzu^®^, Kyoto, Japan) under a controlled nitrogen atmosphere with a flow rate of 50 mL·min^−1^. Samples of 3 mg were analyzed in aluminum pans over a temperature range of 25 to 200 °C, using a heating rate of 10 °C·min^−1^.

Thermogravimetric analysis (TGA) was performed using a DTG-60H instrument (Shimadzu^®^, Kyoto, Japan) in platinum pans under a nitrogen atmosphere with a flow rate of 50 mL·min^−1^. Samples ranging from 3 to 5 mg were analyzed over a temperature range of 25 to 500 °C at a heating rate of 10 °C·min^−1^. TGA data were interpreted using the first derivative thermogravimetric (DrTGA) curves. All thermal data were processed using TA-60 software (Shimadzu^®^, Kyoto, Japan), and the results were plotted using OriginPro 2023b software (OriginLab Corp., Northampton, MA, USA).

### 2.5. Development of NECV

The nanoemulsion was prepared using 15% (*v*/*v*) EOCV, 30% (*v*/*v*) Cremophor^®^ EL (Merck, Darmstadt, Germany), 5% Plurol^®^ Oleique (Gatefossè, Saint-Priest, France), and 50% (*v*/*v*) pH 5.5 phosphate buffer. The preparation of the nanoemulsion was carried out based on the method described by Cardoso et al. [26], with adaptations. The EOCV was used as the oil phase at a concentration of 15% (*v*/*v*), which was above the MBC for all tested microorganisms. Cremophor^®^ EL is a hydrophilic surfactant with high emulsifying capacity [27] and was combined with Plurol^®^ Oleique, a lipophilic co-surfactant that contributes to increasing the solubility of hydrophobic drugs [28]. The combination of surfactant and co-surfactant reduces interfacial tension, improving the stability and solubility of nanoemulsion. Furthermore, both are nonionic surfactants, an important feature that minimizes the risk of skin irritation [29]. The mixture was homogenized using an ultrasonic processor (Vibra-Cell VC 750, Sonics & Materials, Inc., Newtown, CT, USA) and sonicated for 20 min at an amplitude of 20% and each cycle consisted of 30 s pulses on and 30 s pulses off. During the process, the temperature was controlled using ice around the beaker. Finally, the NECV was centrifuged at 4000 rpm for 10 min to ensure no phase separation occurred.

### 2.6. Characterization of NECV

The droplet size, polydispersity index (PDI), and zeta potential of the NECV (diluted at a ratio of 1:1000 in ultrapure water) were analyzed using a Zetasizer Nano ZS (Malvern, Worcestershire, UK). To assess the spreadability, an aliquot of 1.0 g of the NECV was placed onto a glass plate, followed by the positioning of a second glass plate over it. A 200 g load was applied to the upper plate for 5 min, after which the diameter of the circle formed by the formulation was measured. Additionally, the pH was measured with a digital pH meter (Digimed, model DM-22, São Paulo, SP, Brazil) by directly immersing the electrode in the undiluted nanoemulsion at 25 ± 1 °C.

For the rheological analysis of NECV, flow behavior was evaluated using a Discovery HR-2 rheometer (TA Instruments, New Castle, DE, USA) equipped with a 50 mm diameter cone-and-plate geometry (1° angle) and a 100 µm gap. Samples were carefully applied to the lower plate of the rheometer to minimize formulation shearing. All measurements were performed after a 5 min thermal equilibration period prior to analysis. Analyses were conducted in triplicate. The flow properties of the gel-based NECV samples were determined at 25 °C over a shear rate range of 0.1–10 s^−1^ [30].

### 2.7. Fourier-Transform Infrared (FTIR) Analysis of EOCV and NECV

A Fourier-transform infrared (FTIR) spectrophotometer, model IR Prestige-21 (Shimadzu, Japan), was used to obtain the spectra of EOCV and NECV, as well as to investigate the involvement of functional groups in the stabilization of the nanoemulsion. FTIR analyses were performed at room temperature. The spectra were recorded in the wavenumber range of 400–4000 cm^−1^, using 80 scans and a spectral resolution of 2.0 cm^−1^.

### 2.8. Short-Term Stability Study of NECV

The short-term stability of the NECV was evaluated for 60 days. The samples stored in hermetically sealed 15 mL Falcon tubes were maintained at 4 ± 2° C and at room temperature (25 ± 2° C) with no humidity control. At time intervals of 1, 9, 15, 30, and 60 days of storage, the samples were tested for droplet size, PDI, zeta potential, and pH. The NECV was also analyzed by Transmission Electron Microscopy (TEM) (model JEM 1011, JEOL, Tokyo, Japan) at time intervals of 1 and 60 days of storage.

For TEM analysis, the sample was diluted (1:1000 *v*/*v*) in ultrapure water, placed on copper grids to be covered with formvar resin, and left to dry at room temperature. After removing the excess with filter paper, the sample was stained with 3 µL of 3% (*w*/*v*) uranyl acetate and left to dry for 3 min at room temperature, protected from light. The excess solution was removed with filter paper, and the sample was analyzed using TEM. The nanostructures were analyzed at magnifications of up to 30,000 times.

### 2.9. HET-CAM Irritation Assay of NECV

The irritation level of NECV was tested using a modified version of the Hen’s Egg Test on the Chorioallantoic Membrane (HET-CAM) [26]. This qualitative method assesses the irritancy potential of drug formulations. The chorioallantoic membrane responds to injury with an inflammatory process similar to that in the rabbit eye’s conjunctival tissue. Fertilized hen’s eggs were obtained from a poultry farm and used on the 9th day of fertilization. Thus, each eggshell was opened to expose the CAM, and a saline solution was applied to hydrate the CAM and the excess was removed. A 1 mol/L NaOH solution was used as positive control and the saline solution as the negative control. In addition to the NECV, a control nanoemulsion containing mineral oil (NOM) (without *C. verbenacea* essential oil) was also evaluated. Then, 300 μL of each sample (NECV, NOM, positive control and negative control) was applied directly to the CAM. After 30 s, the membrane was washed with saline solution to remove the tested products. Then, the CAM was observed for 5 min for the appearance of physiological signs of irritation such as hyperemia, hemorrhage, and coagulation. The time required to cause any of those physiological signs of irritation was totaled to give a single numerical value indicating the irritation potential of the test samples on a scale with a maximum value of 21 [31].

### 2.10. In Vitro Skin Permeation Assay of NECV

The in vitro skin permeation assay was performed using vertical Franz-type diffusion cells assembled with pieces of skin obtained from porcine ears separating the donor compartment from the receptor compartment. The skin was obtained from a local slaughterhouse that sacrifices the animal for food consumption, so it did not require ethical authorization.

The donor compartment was filled with 1 g (i) NECV or (ii) a control formulation composed of 15% OECV and 85% mineral oil. The receptor compartment was filled with 0.01 mol/L phosphate buffer (pH 7.4) containing 5% (*w*/*v*) Tween^®^ 20 to ensure sink conditions. The receptor medium was kept under constant magnetic stirring at 300 rpm and maintained at a temperature of 35 ± 2 °C for 24 h. After the incubation period, 1 mL aliquots of the receptor medium were collected and stored in amber vials for later analysis using high-performance liquid chromatography (HPLC) to quantify α-pinene content. The skin was then carefully removed from the diffusion cells, gently cleaned with gauze and purified water, and placed *Stratum corneum*-side up on a flat surface. The skin was then subjected to 15 cycles of adhesive tape stripping to sequentially remove the *Stratum corneum* layers. The collected tapes were immersed in 5 mL of methanol and agitated for 24 h to extract the α-pinene. The remaining skin tissue was cut into smaller fragments to maximize surface area and submitted to methanol extraction for 24 h. The concentrations of α-pinene in both the receptor medium and the skin extracts were quantified by HPLC to assess the permeation efficiency and cutaneous retention.

### 2.11. Quantification of α-Pinene

The α-pinene was quantified considering that *C. verbenacea* essential oil has a proportion of 33.05% of this component. The α-pinene was quantified using a Shimadzu LC 20-AD HPLC system equipped with two LC 20-AT pumps, a SIL-20AD autosampler, a CTO-20AS column oven, and an SPD-M20A diode array detector. Data acquisition and analysis were performed using Shimadzu LC Solutions software (Version 5.127). Separation was achieved on a C18 reversed-phase column (250 mm × 4.6 mm, 5 µm particle size). The mobile phase consisted of ultrapure water and acetonitrile (20:80, *v*/*v*) delivered isocratically at a flow rate of 1.0 mL/min. Samples (20 µL injection volume) were analyzed at 40 °C, with detection set to 210 nm.

The HPLC method for the quantification of α-pinene was validated in accordance with the International Council for Harmonization guideline ICH Q2(R1). Linearity was evaluated using an external standard calibration curve prepared from α-pinene standard solutions in the concentration range of 0.5–20.0 µg/mL. The curve was constructed by plotting peak area versus nominal concentration and applying linear regression analysis. Precision was assessed in terms of repeatability through replicate injections of standard solutions at different concentration levels within the calibration range under identical chromatographic conditions and was expressed as relative standard deviation (RSD). The limits of detection (LOD) and quantification (LOQ) were determined based on the standard deviation of the analytical response and the slope of the calibration curve, following the mathematical approach recommended by ICH Q2(R1). Method specificity was evaluated by comparing chromatograms obtained from α-pinene standard solutions, solvent blanks, and sample matrices to confirm the absence of interfering peaks at the analyte retention time. This validation protocol ensured the reliability of the method for the quantitative analysis of α-pinene.

### 2.12. Statistical Analysis

All analyses were performed in triplicate, and the results are expressed as the mean ± standard deviation (SD). Data was treated with the aid of STATISTICA software version 10.0. Analysis of variance (ANOVA) was performed to detect significant differences between the analyses, and when differences were statistically significant, Tukey’s test for mean comparisons was used.

## 3. Results and Discussion

### 3.1. Chemical Composition of EOCV

The analysis of the chemical composition of the EOCV revealed the presence of 40 volatile compounds, accounting for 97.24% of the total composition (Table 1). The sesquiterpenes constituted the predominant class (60.58%), followed by monoterpenes (36.66%). The most abundant individual components were the monoterpene α-pinene (33.05%) and the sesquiterpene β-caryophyllene (25.11%). Other components present in considerable amounts included the sesquiterpenes δ-elemene (4.97%), α-humulene (4.18%), and allo-aromadendrene (6.80%).

Other studies in the literature also have reported α-pinene (27.1–58.9%) and β-caryophyllene (12.5–23.9%) as the terpenes with the highest proportions in the EOCVs obtained from different regions of Brazil (Campinas, São Paulo State, Southeast region; Crato, Ceará State, Northeast region; and Parnaíba, Piauí State, North region). The terpenes α-humulene (3.43–4.17%) and allo-aromadendrene (4.37–8.60%) were also identified in the different EOCVs [3,4,32]. Silva et al. [33] reported that the chemical analysis of seven genotypes of EOCVs from São Cristóvão, Sergipe State, in the Northeast region of Brazil, revealed the presence of β-caryophyllene (1.46–21.78%) and α-humulene (0.61–4.98%) in their composition. The compounds α-humulene and β-caryophyllene are considered chemical markers of EOCV, and β-caryophyllene has been reported in the literature to exhibit anti-inflammatory and antimicrobial effects [34,35].

Marques et al. [34] described the chemical profiles of EOCVs from São Paulo (SP) and Rio de Janeiro (RJ), in the Southeast region of Brazil, according to the seasons of the year. The compound with the highest proportion, regardless of the season, was α-pinene, ranging from 42.8 to 60.0% for the EOCV from RJ and from 14.6 to 38.9% for the EOCV from SP. The EOCV from RJ showed the highest α-humulene content, with greater production during the spring (2.89%), while the EOCV from SP exhibited the highest β-caryophyllene content (16.12–17.20%) across all evaluated seasons. Fonseca et al. [36] observed that the α-humulene content of the EOCV from Oratórios, MG, in the Southeast region of Brazil, was within the recommended standards (2.30 to 2.90%) during all evaluated seasons: summer (4.91%), autumn (4.89%), and winter (3.90%). The essential oil yield was significantly higher in winter, probably due to the dry season.

Table 2 presents the content of phenolic compounds and antioxidant activity determined by DPPH and ABTS methods for the EOCV. The content of phenolic compounds presented by EOCV (1.20 mg/mL) was comparable to that of other essential oils, such as oregano (1.93 mg/mL, *Origanum vulgare*), thyme (1.63 mg/mL, *Thymus vulgaris*) [37], and lavender (0.52 mg/mL, *Lavandula spica*) [38]. Phytochemical studies conducted on *C. verbenacea* leaves identified the presence of flavonoids (quercetin, rutin, 7,4′-dihydroxy-5′-carboxymethoxy isoflavone, 7,4′-dihydroxy-5′-methyl isoflavone, brickellin, and artemetin) and phenolic acids (rosmarinic acid, gallic acid, chlorogenic acid, and caffeic acid) [39]. Camargo et al. [40] reported that the extracts of *C. verbenacea* leaves exhibited total phenolic contents ranging from 15.59 to 49.36 mg/mL.

The antioxidant potential of an EO can be predicted by considering its composition and in general, a good antioxidant property is expected if the EO contains high amounts of phenolic compounds [37]. The antioxidant activity presented by EOCV was 915.32 and 102.47 μmol TE/mL by the ABTS and DPPH methods, respectively. Mutlu-Ingok et al. [37] reported that the essential oils of thyme and oregano, which have recognized antioxidant activity, showed results of 634 and 122 μmol TE/mL, respectively, as assessed by the CUPRAC method. Camargo et al. [40] reported that EOCV exhibited an IC_50_ value of 49.58 mg/mL using the DPPH method. Compounds with antioxidant activity are known for their ability to neutralize free radicals and prevent oxidative stress in cells. This effect occurs through the donation of hydrogen or electrons, stabilizing the radicals and interrupting chain reactions that could lead to cellular damage [40].

The ability to neutralize free radicals is one of the most attractive biological properties of natural products. Containing a mixture of lipophilic antioxidant compounds, essential oils have been employed in formulations as preservatives to delay oxidation and extend the shelf life of nanoemulsions [41]. In addition, in both acute and chronic wounds, elevated levels of ROS (Reactive Oxygen Species) damage cells, proteins, and extracellular matrices, thereby delaying re-epithelialization and collagen deposition. Antioxidants neutralize ROS, protecting keratinocytes and fibroblasts and promoting cell proliferation and migration [42]. Thus, products with antioxidant capacity reduce pro-inflammatory markers (TNF-α, IL-6) and/or activate protective pathways (such as Nrf2, nuclear factor erythroid–related factor 2), which helps to limit chronic inflammation and facilitates the transition to the proliferative phases of wound healing [43].

### 3.2. Determination of Minimal Inhibitory Concentration (MIC) and Minimal Bactericidal Concentration (MBC) of EOCV

The MIC and MBC values of EOVC were lower for the yeasts *C. albicans* and *C. krusei* (6.3 to 25.0 µL/mL) and for Gram-positive *B. cereus*, *S. aureus* and *E. faecalis* (11.3 to 25.0 µL/mL) compared to the Gram-negative *A. baumannii*, and *K. pneumoniae E. coli* (12.5 to 75.0 µL/mL) (Table 3).

Camargo et al. [40] obtained similar results and reported that the EOCV from Rio Grande do Sul State, southern Brazil, exhibited MIC values of 6.25 mg/mL for *S. aureus* and *E. faecalis*, and MBC values of 6.25 mg/mL for *S. aureus* and 12.5 mg/mL for *E. faecalis*. However, no antimicrobial activity was observed at the tested concentrations against the Gram-negative bacteria *Salmonella choleraesuis* and *E. coli*.

The EOVC contains a significant amount of monoterpenes and sesquiterpenes, which are the main compounds responsible for its antimicrobial activity, especially against Gram-positive bacteria and yeasts [1]. Gram-negative bacteria are less sensitive to EO due to their hydrophilic outer membrane, which acts as a barrier limiting the diffusion of active compounds, making these bacteria more resistant [44].

In the present study, the EOCV showed the lowest MIC (6.30 µL/mL) and MFC (12.5 µL/mL) values for the yeast *C. albicans*. Other studies in the literature have also reported antifungal activity for the Brazilian EOCV. Nizio et al. [45] observed a fungicidal effect of EOCV produced from leaves collected in Sergipe State, northeastern Brazil and at concentrations lower than 100 µL/mL, there was inhibition of the mycelial growth of the fungus *Lasiodiplodia theobromae*. Camargo et al. [40] reported that the EOCV from Rio Grande do Sul State, southern Brazil, exhibited inhibition halos ranging from 7.5 to 12.0 mm using the disk diffusion method against the fungi *Penicillium crustosum*, *Aspergillus flavus*, and *Alternaria alternata*. Silva et al. [46] reported that the combination of EOCV from Ceará State, Northeast Brazil with fluconazole significantly reduced the MIC required for the drug to inhibit *C. albicans* and *C. krusei*.

The MIC and MBC values of the EOCV were comparable to those of other essential oils reported in the literature. The MIC (13.0 µL/mL) and MBC (25.0 µL/mL) values of EOVC for *S. aureus* were similar to those of sage (*Salvia officinalis*) and spearmint (*Mentha spicata*) essential oils, which showed for *S. aureus* MICs of 12.5 µL/mL and MBCs of 25.0 µL/mL [47]. The Gram-negative bacterium *E. coli* presented a MIC value (13.0 µL/mL) for EOCV, and de Medeiros Barbosa et al. [48] reported for rosemary (*Rosmarinus officinalis*) essential oil values of MIC of 5.0 µL/mL for *E. coli* and 10.0 µL/mL for *Salmonella Enteritidis*. For the yeast *C. albicans*, EOCV showed MIC of 6.30 µL/mL and MFC of 12.5 µL/mL and Almeida et al. [49] reported higher values for *C. albicans* when using citronella (*Cymbopogon winterianus*) and cinnamon (*Cinnamomum cassia*) essential oils, with respective MICs of 65.0 and 250.0 µL/mL, which were also their MFC values.

The antimicrobial mechanisms of EO generally involve interference with functional components of the membrane, cell wall, cell replication and protein synthesis. EO can penetrate and destabilize the lipid bilayer of microbial cell membranes, increasing membrane permeability. This leads to leakage of vital intracellular contents such as ions, ATP, and nucleic acids, ultimately resulting in cell death. Some EO components may compromise the integrity of the microbial cell wall, especially in Gram-positive bacteria, which are more susceptible due to their simpler wall structure. EO constituents can bind to microbial enzymes or proteins, altering their structure and inhibiting their function. This affects essential metabolic pathways, such as energy production or DNA replication [50,51].

### 3.3. Compatibility Studies

**Figure 1:**
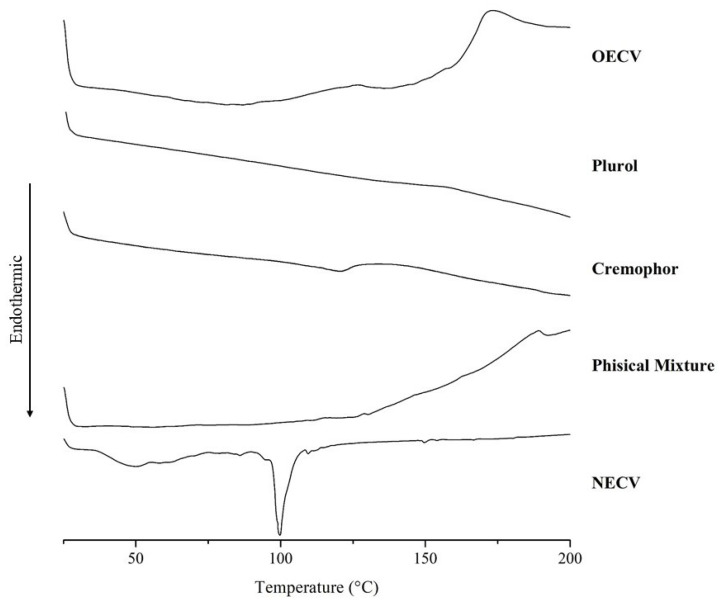
Differential scanning calorimetry (DSC) of the isolated components (EOCV, Cremophor and Plurol), the physical mixture of the components, and the nanoemulsion formulation (NECV).

The thermal analysis of the samples using differential scanning calorimetry (DSC) (Figure 1) was performed to investigate the thermal behavior of the formulation components and potential physicochemical interactions.

For the physical mixture composed of EOCV, Plurol, and Cremophor, the thermogram did not exhibit evident endothermic or exothermic events at intermediate temperatures, indicating the absence of detectable phase transitions within the analyzed range. However, an exothermic event was observed at the end of the test, with a peak temperature (T_peak_) at 189.01 °C, which can be attributed to the thermal decomposition of the essential oil, suggesting its thermal instability at elevated temperatures.

In contrast, the DSC curve of the NECV showed two distinct thermal events. The first, characterized as a broad endothermic band, occurred between 35.86 °C and 74.80 °C, with a T_peak_ at 49.54 °C, likely associated with the evaporation of free water within the emulsion matrix. The second, a sharper and more defined endothermic event, was recorded between 91.06 °C and 108.61 °C, with a T_peak_ at 99.74 °C, possibly related to structural alterations in the surfactants (Plurol and Cremophor), along with the release and volatilization of essential oil components present in the nanoemulsion system. These results suggest that incorporating the essential oil into a nanoemulsion system modifies its thermal behavior, conferring relative stability and allowing the identification of thermal events not detectable in the physical mixture.

**Figure 2:**
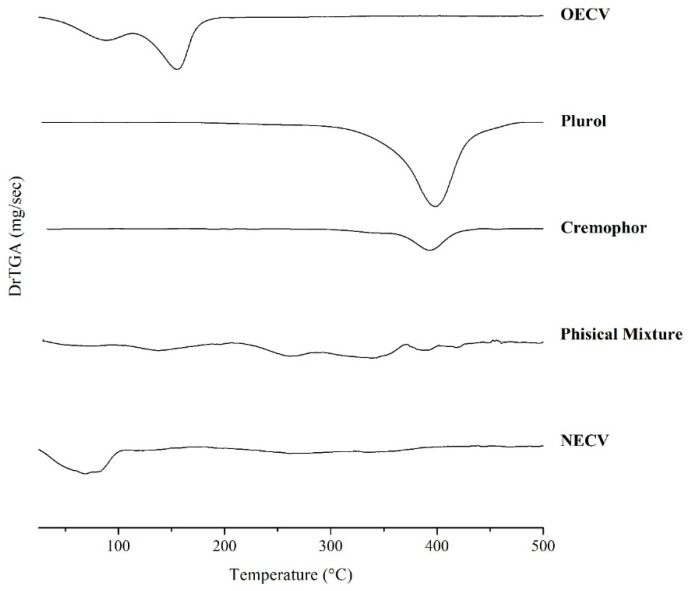
First derivative of the thermogravimetric analysis (DrTGA) of the isolated components (EOCV, Cremophor and Plurol), the physical mixture of the components, and the nanoemulsion formulation (NECV).

In the derivative TGA (DrTGA) curves (Figure 2), the results demonstrated distinct thermal behaviors among the analyzed materials. The EOCV showed a decomposition profile with two well-defined thermal stages. The first stage occurred between 30.36 °C and 112.83 °C, with a decomposition T_peak_ at 88.88 °C and a mass loss of 34.56%, attributed to the evaporation of more volatile constituents. The second stage, from 112.83 °C to 193.30 °C (T_peak_ 155.09 °C), resulted in an additional mass loss of 57.63%, indicating the thermal degradation of less volatile fractions of the EOCV.

The surfactant Plurol exhibited a single decomposition event, with a temperature range from 290.55 °C to 483.38 °C and a maximum T_peak_ at 398.13 °C, corresponding to a mass loss of 94.71%, indicating high thermal stability up to approximately 290 °C. Similarly, the surfactant Cremophor displayed a single thermal event between 354.44 °C and 440.40 °C, with a T_peak_ at 392.32 °C and a mass loss of 80.03%, showing comparable thermal stability, although slightly lower than that of Plurol.

The physical mixture of the components did not show well-defined thermal decomposition events, reflecting the complexity and potential interactions among the constituents. Three possible decomposition stages were identified: the first between 31.43 °C and 205.67 °C (mass loss of 27.10%), the second between 205.67 °C and 370.61 °C (mass loss of 50.95%), and the third between 370.61 °C and 500 °C (mass loss of 17.09%). This behavior may indicate the overlapping of thermal events from the individual components.

Finally, the NECV exhibited a distinct thermal profile, characterized by a prominent decomposition stage between 26.78 °C and 108.09 °C, with a T_peak_ for mass loss at 68.24 °C and a variation of 49.60%, likely related to the evaporation of water molecules and volatile compounds from the essential oil. The second, less prominent phase of mass loss occurred between 179.96 °C and 437.52 °C, with an additional loss of 38.35%, corresponding to the thermal degradation of excipients and possibly less volatile fractions of the essential oil incorporated into the nanoemulsion structure.

Thus, the TGA results demonstrate that incorporating the EOCV into a nanoemulsion influences the system’s thermal stability, altering the mass loss profiles and decomposition events compared to the isolated components or their physical mixture. However, the interpretation of formulations containing lipids in their composition is highly complex, and multiple physicochemical evaluations are necessary to provide a more accurate assessment of the compatibility between the components in the nanoemulsion system [26].

### 3.4. Characterization and Stability of NECV

**Figure 3:**
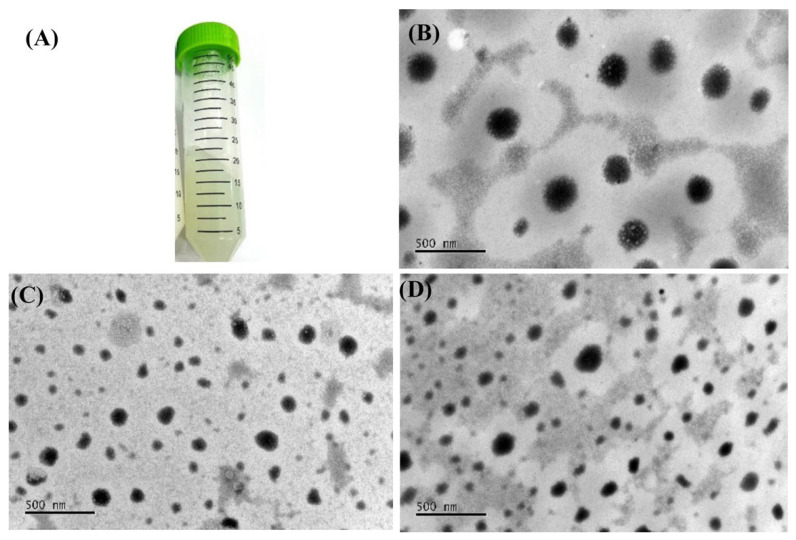
(**A**) Appearance of the *C. verbenacea* essential oil nanoemulsion (NECV), (**B**) TEM morphology of the NECV on the first day after formulation, (**C**) TEM morphology of the NECV on the 60th day of storage at 25 °C, (**D**) TEM morphology of the NECV on the 60th day of storage at 5 °C.

The NECV was transparent with a greenish hue and exhibited high viscosity, as shown in Figure 3. After preparation, the NECV was subjected to centrifugation at 4000 rpm for 10 min, and no phase separation was observed.

Nanoemulsions (NEs) are colloidal dispersions with droplet sizes in the nanometric range, typically between 20 and 500 nm [52]. The NECV showed a droplet size of 126.80 nm, which is consistent with the definition of a nanoemulsion (Table 4). The small droplets of nanoemulsion facilitate particle penetration and exhibit good resistance to aggregation [41]. The polydispersity index (PDI), which ranges from 0 to 1, reflects the particle size distribution in suspensions, with a lower PDI indicating a more uniform size distribution [53]. The PDI of NECV was 0.49, indicating good dispersion and a relatively uniform particle size distribution.

Other studies on essential oil NEs have shown similar results for droplet size and PDI. Li et al. [53] obtained a nanoemulsion of *Zanthoxylum bungeanum* essential oil with a particle size of 134.37 nm and a PDI of 0.19. Yan et al. [41] reported that *Camphora longepaniculata* essential oil NEs had an average droplet size of 74.90 nm and a PDI of 0.42.

The NECV exhibited a zeta potential of −18.50 mV. Zeta potential refers to the electric charge on the droplets’ surface and assesses their ability to remain suspended, i.e., without aggregating or settling [52]. The choice of emulsifier significantly influences the zeta potential of a nanoemulsion. Non-ionic emulsifiers, such as those used in the present study (Cremophor^®^ EL and Plurol^®^ Oleique) are compounds that reduce interfacial tension between the oil and aqueous phases, thereby preventing droplet aggregation. These emulsifiers rapidly adsorb at the oil-water interface, conferring both electrostatic and steric stability [54]. Zhao et al. [55] reported that oregano essential oil NEs stabilized with the non-ionic surfactant Tween 80 exhibited zeta potential values ranging from −15.00 to −18.10 mV. Although Tween 80 is a non-ionic surfactant, the nanoemulsion showed a negative zeta potential due to the ability of hydroxyl ions to be adsorbed from water onto the surface of the oil droplets.

The NECV was formulated using a phosphate buffer with a pH of 5.5, and the resulting pH after formulation was 5.3. The pH of the human *Stratum corneum* ranges from 4.2 to 5.6 on the surface [55,56], so the pH nanoemulsion may be compatible with the skin. Yan et al. [41] reported similar results to *Camphora longepaniculata* essential oil NE that had a pH of 5.4.

The spreadability of NECV was 4.70 cm, and the formulation exhibited good spreading ability. Alhasso et al. [57] reported lower spreadability values of 3.70 and 3.80 cm for mupirocin-loaded nanoemulgels formulated with eucalyptus essential oil and eucalyptol, respectively. Kesharwani et al. [58] obtained a spreadability value of 6.00 cm for a curcumin-loaded nanogel intended for topical application and concluded that the application of topical preparations becomes easier and more uniform when the formulation displays good spreadability, with values between 5 and 7 cm being indicative of good spreadability.

The morphology of the NECV droplets was observed under TEM, as shown in Figure 3. On the first day after formulation, the NECV appeared as dispersed small droplets and spherical shape (Figure 3B). The NECV was subjected to stability testing under two conditions: refrigeration (4 ± 2 °C) and ambient temperature (25 ± 2 °C). Figure 3C and Figure 3D, which represent samples stored for 60 days at 25 °C and 4 °C, respectively, demonstrate morphological stability comparable to that observed on day 1, indicating NECV preserves its physical structure over time.

**Figure 4:**
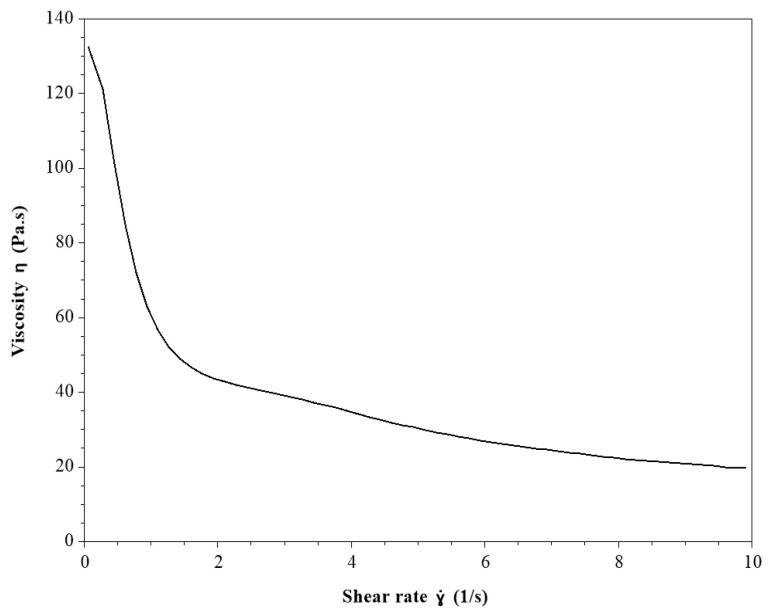
Apparent viscosity (Pa·s) as a function of shear rate (0.1–10 s^−1^) at 25 °C of the nanoemulsion formulation (NECV).

The results of the rheological analysis of NECV showed that the gel exhibited non-Newtonian pseudoplastic (shear-thinning) behavior, characterized by high viscosity at low shear rates and a progressive decrease in viscosity as the shear rate increased, with this reduction becoming less pronounced at higher shear rates (Figure 4).

Shear rate is a key parameter in viscosity measurements, as variations in shear rate lead to changes in viscosity depending on the type of formulation. This property is particularly relevant for topical formulations, as it helps prevent product runoff from the finger or the application site and promotes easy and uniform spreading of the formulation over the skin surface [57].

**Figure 5:**
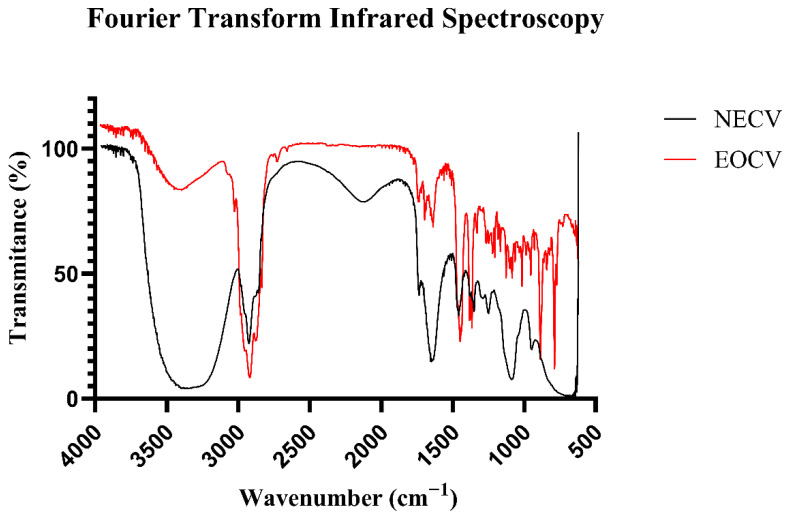
FTIR spectra of *Cordia verbenacea* essential oil (EOCV, red line) and its nanoemulsion (NECV, black line), recorded in the 4000–500 cm^−1^ wavenumber range.

Figure 5 presents the Fourier-transform infrared (FTIR) spectra of EOCV and its nanoemulsion, enabling the identification of functional groups and the assessment of EOCV incorporation into the nanoemulsion system. The FTIR spectrum of the free essential oil (EOCV) exhibited characteristic bands of terpene-rich compounds. A broad absorption band centered at approximately 3400 cm^−1^ was observed, which is attributed to O–H stretching vibrations of hydroxyl groups involved in hydrogen bonding [59,60]. The intense bands at around ~2920 cm^−1^ and ~2850 cm^−1^ correspond to the asymmetric and symmetric C–H stretching vibrations of methylene (CH_2_) and methyl (CH_3_) groups, respectively, which are commonly present in the aliphatic chains of terpenes [59,61]. The presence of carbonyl-containing compounds, such as esters, aldehydes, or ketones, was indicated by the C=O stretching band in the 1740–1700 cm^−1^ region. Absorption bands between 1640 and 1600 cm^−1^ are associated with stretching vibrations of carbon–carbon double bonds (C=C), confirming the unsaturated nature of the terpene constituents of EOCV [59,60].

Modifications in the FTIR spectrum were observed after the incorporation of EOCV into the nanoemulsion (NECV). The O–H stretching band (~3400 cm^−1^) exhibited pronounced broadening and increased intensity, which is attributed to the contribution of the aqueous phase and the hydroxyl groups of the surfactants, as well as to the strengthening of hydrogen-bonding networks within the colloidal system [60,62,63]. The characteristic aliphatic C–H stretching bands (2920 and 2850 cm^−1^) remained present, although with reduced relative intensity, suggesting the preservation of the hydrocarbon structures of EOCV, partially masked by the nanoemulsion matrix [62,63]. Minor shifts or changes in intensity in the carbonyl region (~1740–1700 cm^−1^) were also observed, indicating physical interactions between the constituents of EOCV and the surfactant molecules. The FTIR analysis confirmed that the main functional groups of EOCV remained intact after the nanoemulsification process. The observed changes are consistent with physical encapsulation and interactions between the oil and surfactant molecules within the nanoemulsion, supporting the successful incorporation of the oil into the carrier system [62,63,64].

**Figure 6:**
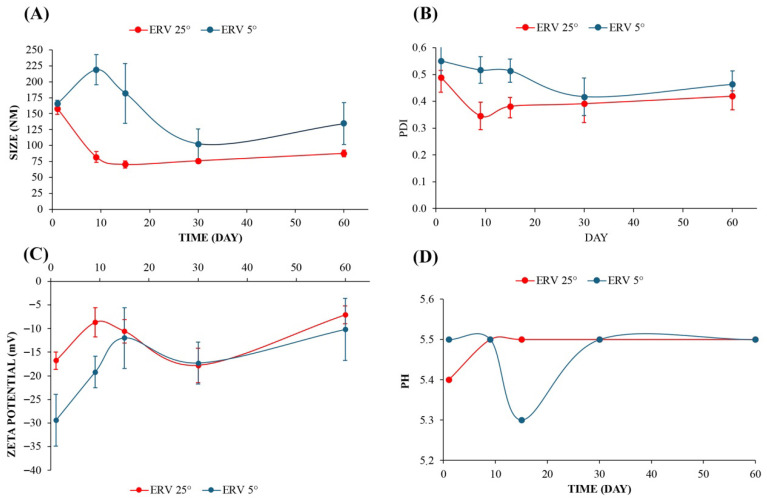
Effects of storage time (1–60 days) at 25 °C and 4 °C on the droplet size (**A**), PDI (**B**), zeta potential (**C**), and pH (**D**) of the nanoemulsion of *Cordia verbenacea* essential oil. The bars represent the standard deviation of determinations.

The variations in droplet size, polydispersity index (PDI), zeta potential, and pH values during the stability study of NECV stored for 60 days at 25 °C and 4 °C are presented in Figure 6. The mean droplet sizes values of NECV remained below 200 nm throughout the 60-day storage period at both temperatures of 25 °C and 4 °C. On the first day of storage, droplet sizes were similar between the different storage temperatures (157.9 nm at 25 °C and 157.6 nm at 4 °C). However, between the 9th and 15th days of storage, the refrigerated samples showed larger droplet sizes (198.1–181.9 nm) compared to those stored at room temperature (80.0–68.8 nm). From the 30th day onward, droplet sizes in the refrigerated samples (98.9 nm) and those stored at room temperature (75.1 nm) became more similar, exhibiting stable values through the 60th day (91.8 nm at 4 °C and 87.7 nm at 25 °C). Droplet size is an important parameter for evaluating the stability of nanoemulsions, with smaller droplet sizes appearing to be one of the most relevant factors in stabilizing these dispersions [52]. Thus, the small droplet size in nanoemulsions helps prevent coalescence and precipitation, which in turn enhances system stability and minimizes degradation over time [65].

The mean PDI values of NECV on the first day of storage were similar between the different storage temperatures (0.47 at 25 °C and 0.54 at 4 °C). However, the samples stored at room temperature (0.34–0.41) exhibited lower PDI values between days 9 and 15 of storage compared to the refrigerated sample (0.54–0.50). Between days 30 and 60 of storage, PDI values (0.39–0.42 at 25 °C and 0.46–0.47 at 4 °C) stabilized in both samples. The polydispersity index (PDI) serves as an indicator of emulsion stability, as it is directly correlated with the uniformity of droplet sizes. A lower PDI value indicates a more uniform droplet size distribution and greater emulsion stability. In emulsions where coalescence and flocculation occur, droplet sizes vary widely, resulting in a higher PDI. Monitoring changes in PDI over time can provide valuable insights into the performance and stability of the emulsion [65].

The zeta potential is another fundamental parameter for the thermodynamic stability of emulsions [51]. A high zeta potential value (whether positive or negative) indicates strong electrostatic repulsion between droplets, preventing them from coming close enough to aggregate or coalesce. Conversely, a low zeta potential, especially near zero, reflects weak electrostatic repulsion, making the nanoemulsion unstable [65]. During the storage of NECV, between days 1 and 9, the zeta potential of the refrigerated sample (−29.1 and −18.9 mV) was higher than that of the sample stored at room temperature (−16.5 and −13.1 mV). However, from the 15th day of storage onward, the zeta potential values of the refrigerated (−15.8 mV) and room temperature (−14.8 mV) samples became more similar, showing average values of −21.0- mV at 30 days of storage and −19.4 mV at 60 days of storage (refrigerated sample) and −20.6 at 30 days of storage and −17.6 mV at 60 days of storage (room temperature sample).

During the 60-day storage of NECV at 25 °C and 4 °C, the pH values remained constant (5.3–5.5) throughout the study, in accordance with the physiological pH of the skin (4.2–5.6), which reinforces the formulation’s suitability for topical applications.

Thus, the NECV remained stable over 60 days of storage at both refrigerated and room temperatures, presenting a droplet size of 87.7–91.8 nm, a PDI of 0.42–0.47, a zeta potential of −19.4 to −17.6 mV, and a pH of 5.3. Similar findings have been reported in the literature for nanoemulsions prepared with other essential oils, which also maintained physical stability during storage under refrigeration and at room temperature [55,66].

Long-term stability studies of NECV (6 to 12 months) are necessary and should additionally include stress condition evaluations (centrifugation, freeze–thaw cycles, and light exposure) to support the long-term stability of the nanoemulsion system.

### 3.5. HET-CAM Irritation Assay of NECV

The hen’s egg test on the chorioallantoic membrane (HET-CAM) is a simple, low-cost in vitro model that serves as an alternative to the use of animals, capable of evaluating the irritating potential of substances and formulations. The HET-CAM test is a widely used assay to assess the irritability of formulations administered via the ocular route and can also be used to evaluate the irritant potential of dermatological formulations [67]. It is based on the identification of irritation reactions following contact with the formulation, which can be classified as hemorrhage, lysis, and coagulation of the chorioallantoic membrane of the fertilized chicken egg [68].

**Figure 7:**
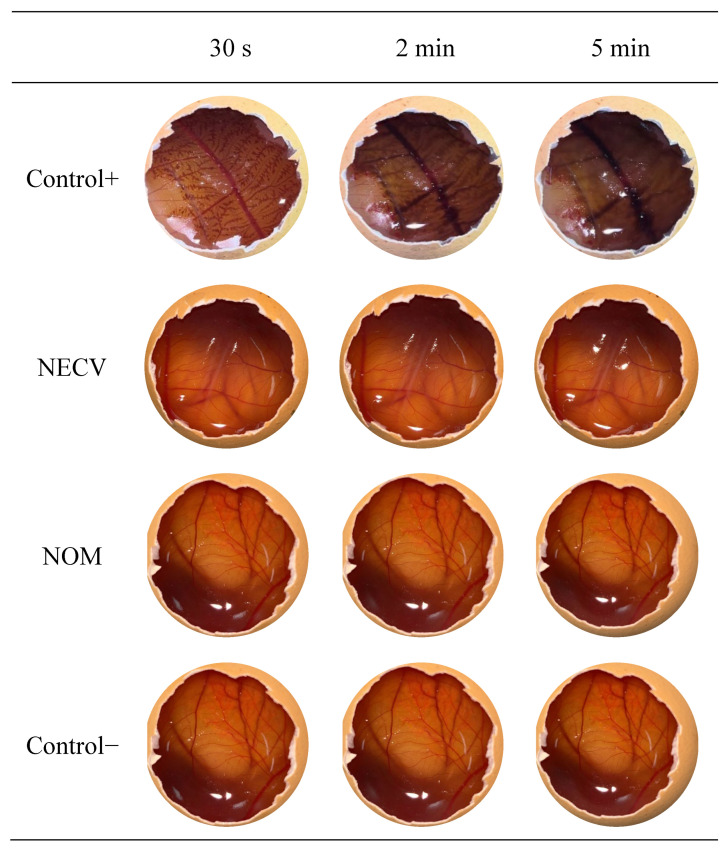
Hen’s egg test on chorioallantoic membranes (HET-CAM) treated with positive (NaOH 1 mol/L) and negative (saline solution) controls, as well as with the nanoemulsion of *C. verbenacea* essential oil (NECV) and the control nanoemulsion containing mineral oil (NOM), at 30 s, 2 min, and 5 min.

Emulsions contain surfactants in their composition, which may potentially cause skin irritation [69]. Therefore, the HET-CAM assay was performed with the nanoemulsion of *C. verbenacea* essential oil (NECV) and the control nanoemulsion containing mineral oil (NOM) (without *C. verbenacea* essential oil), and images of the CAM membranes after 30 s, 2 min, and 5 min of exposure to each formulation are shown in Figure 7.

There was no vessel rupture or blood leakage (hemorrhage, lysis, or coagulation) with the negative control (saline solution), whereas the positive control (1 M NaOH) caused these effects within the first few seconds of exposure, being classified as extremely irritating. The NECV and NOM formulations showed responses similar to the negative control, with no signs of vascular damage. After 5 min of observation, the irritation score (IS) was calculated as 19 for the positive control, 0 for the negative control, 0 for NECV, and 0 for NOM. These results classify both nanoemulsions as non-irritating, confirming that the surfactants and the *C. verbenacea* essential oil did not cause irritating effects. This finding reinforces the potential use of NECV for topical application, offering anti-inflammatory and antimicrobial effects without the risk of causing skin irritation.

The results obtained in the test are similar to those reported in the literature for other micro- and nanoemulsions. Studies evaluating natural products for topical or mucosal application have reported low irritation potential. Microemulsions with *Brosimum gaudichaudii* extracts, nanoemulsions containing *Piper aduncum* essential oil, and hydrogels incorporating nanoemulsions of *Pelargonium graveolens* essential oil were all classified as slightly irritating or non-irritating and considered safe for use [67,68,69].

### 3.6. In Vitro Skin Permeation Assay of NECV

**Figure 8:**
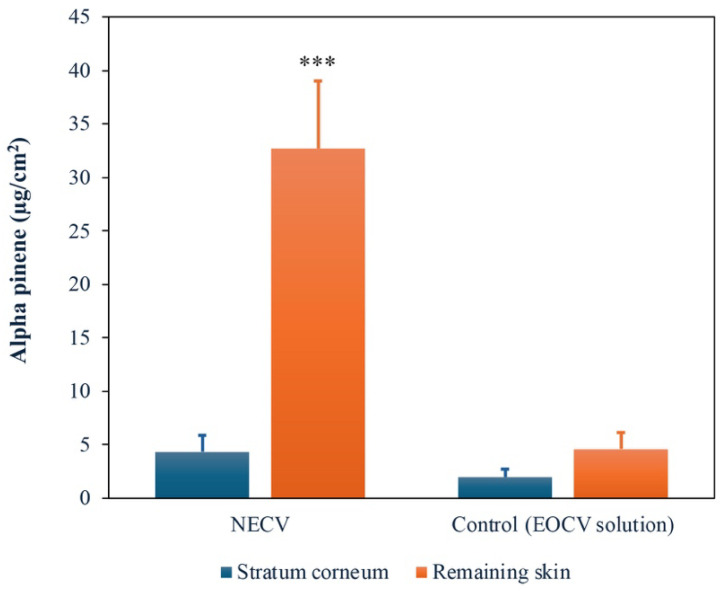
Alpha-pinene penetration in skin layers (the *Stratum corneum* and remaining skin) after 24 h of NECV and a non-nanoemulsified control (15% of free essential oil in mineral oil) topical application. The bars represent the standard deviation of determinations. Asterisks indicate a significant difference between NECV and control with *p* value 0.0001 (considering *p* value < 0.05 according to the Tukey test at the 95% confidence level).

Figure 8 presents the permeation of alpha-pinene across the *Stratum corneum* and remaining skin, which comprises the viable epidermis and part of the dermis, after 24 h of NECV topical application compared to a control oil solution.

**Figure 9:**
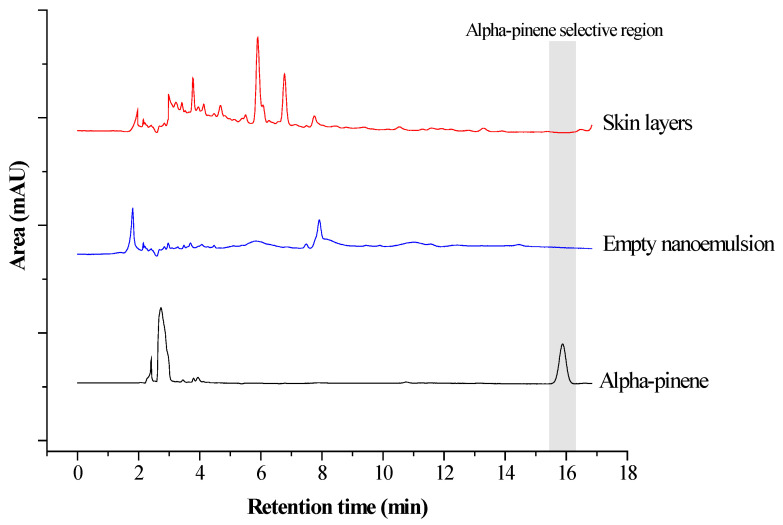
Selective chromatographic profile of α-pinene in standard solution, empty nanoemulsion, and skin samples.

The HPLC method for the quantification of α-pinene was validated in accordance with the ICH Q2(R1) guidelines. Linearity was evaluated over the concentration range of 0.5–20.0 µg/mL, showing excellent correlation between concentration and peak area (R^2^ = 0.9997). Method precision was assessed in terms of repeatability, with relative standard deviation (RSD) values ranging from 2.69% to 5.52%, which are within acceptable limits for analytical methods. The limit of detection (LOD) and limit of quantification (LOQ), calculated based on the standard deviation of the response and the slope of the calibration curve, were 0.40 µg/mL and 1.22 µg/mL, respectively, indicating adequate method sensitivity. Method specificity was confirmed by the absence of interfering peaks at the retention time of α-pinene, as demonstrated by representative chromatograms of the standard solution, solvent blank, and sample matrix (Figure 9). These results confirm that the proposed HPLC method is linear, precise, sensitive, and specific for the quantitative determination of α-pinene.

The selection of α-pinene as a permeability marker was based on its high concentration (33.05%) in the *C. verbenacea* essential oil. The monoterpene α-pinene exhibits antibacterial and antifungal activity against several microbial species, and its mechanism of action involves destabilization of microbial cell membranes, modulation of efflux pump activity in bacteria, thereby increasing cell permeability, and induction of oxidative stress [70,71]. Another marker that could have been used is β-caryophyllene, which was present at a concentration of 25.11% in the *C. verbenacea* essential oil. This sesquiterpene displays antibacterial and anti-inflammatory properties and has been investigated as a skin permeation enhancer in topical formulations [72,73]. Together, these findings suggest a synergistic or complementary role between α-pinene and β-caryophyllene in topical formulations: α-pinene providing primary antimicrobial membrane disruption, while β-caryophyllene potentially enhances the skin penetration of active constituents [74,75,76].

The NECV achieved a 7.1-fold greater permeation of the marker alpha-pinene into the deeper layers of the skin (32.7 ± 6.3 μg/cm^2^ vs. 4.6 ± 1.5 μg/cm^2^ for the control; *p* < 0.05), demonstrating its ability to overcome the *Stratum corneum* barrier. Other studies have also demonstrated the ability of micro- and nanoemulsified systems to enhance the skin permeation of natural active compounds. Nanoemulsions of *Piper aduncum* essential oil enabled dermal penetration of dillapiole, with no detectable residue in the *Stratum corneum* after 8 h, indicating deep tissue absorption [68]. Similarly, microemulsions containing *Pouteria macrophylla* fruit extract achieved greater skin retention compared to conventional emulsions, especially in the viable epidermis and dermis (target sites for depigmenting activity) [77]. Finally, the hydrogel containing the nanoemulsion of *Pelargonium graveolens* essential oil showed slight irritation potential, suggesting a safe product for application to the vaginal mucosa [69].

Essential oils and their active compounds can cross the skin barrier by disrupting the intercellular packing of *Stratum corneum* lipids, which may explain their high skin permeation. Nanoemulsions, due to their small droplet size, exhibit a high surface-to-volume ratio, enhancing cellular permeation efficiency and promoting diffusion into deeper epidermal and dermal compartments of the skin [67]. This behavior is particularly desirable, as numerous cell types involved in inflammatory and wound-healing processes are in the dermis.

The results are highly promising regarding the potential application of NECV as a transdermal phytotherapeutic for the treatment of skin wounds, especially in dermatological therapies that require deep-tissue bioavailability of active compounds. Future studies are necessary to evaluate cytotoxicity and to perform in vitro scratch-wound or in vivo assays to confirm the antimicrobial and wound-healing properties of NECV.

## 4. Conclusions

The present study demonstrated that EOCV exhibited antimicrobial activity, showing greater effect against the yeasts *Candida albicans* and *C. krusei*, as well as the Gram-positive bacteria *Bacillus cereus*, *Staphylococcus aureus*, and *Enterococcus faecalis*. The stability study of NECV revealed that the formulation remained stable over 60 days under both room temperature and refrigerated conditions, with a presenting a droplet size of 87.6–91.8 nm, a PDI of 0.42–0.47, a zeta potential of −19.4 mV−17.6 mV, and a pH of 5.3–5.5. Furthermore, in the HET-CAM irritation test, the formulation was classified as non-irritating, and in the in vitro skin permeation assay, NECV stimulated the penetration of the active to the deeper skin layers, demonstrating its ability to overcome the *Stratum corneum* barrier. The results highlight the potential of NECV for topical use, as nanoemulsions can enhance the pharmacological efficacy of EOCV as an antimicrobial agent, making it a promising therapeutic alternative for the treatment of infected skin wounds.

## Figures and Tables

**Table 1:** Chemical composition (%) of *C. verbenacea* essential oil.

	Compounds	RI^a^	RI^b^	Composition (%)
1	α-Thujene	919	924	0.21
2	α-pinene	931	932	33.05
3	α-Fenchene	944	945	0.15
4	Sabinene	962	969	0.51
5	β-Pinene	970	972	0.43
6	Myrcene	981	988	0.23
7	α-Phellandrene	1000	1002	0.06
8	α-Terpinene	1011	1014	0.04
9	o-cymene	1018	1020	0.06
10	Limonene	1024	1024	0.45
11	1,8-Cineole	1025	1026	1.02
12	γ-Terpinene	1051	1054	0.06
13	n-nonanal	1098	1100	0.30
14	α-Campholenal	1120	1122	0.07
15	trans-Sabinol	1132	1137	0.08
16	δ-Elemene	1335	1335	4.97
17	α-Copaene	1369	1374	0.57
18	β-Bourbonene	1383	1387	0.28
19	β-Elemene	1384	1389	2.33
20	Sesquithujene	1401	1405	0.76
21	α-cis-Bergamotene	1408	1411	0.74
22	β-caryophyllene	1413	1417	25.11
23	β-Copaene	1426	1430	0.57
24	α-trans-Bergamotene	1430	1432	0.44
25	6,9-Guaiadiene	1437	1442	0.19
26	α-Humulene	1450	1452	4.18
27	(E)-β-Farnesene	1454	1454	0.86
28	allo-Aromadendrene	1455	1458	6.80
29	δ-Amorphene	1506	1511	0.34
30	γ-Cadinene	1512	1513	1.96
31	Zonarene	1532	1528	0.27
32	γ-Cuprenene	1536	1532	2.18
33	trans-Dauca-4(11),7-diene	1559	1556	0.21
34	Germacrene B	1563	1559	0.86
35	(E)-Nerolidol	1566	1561	1.44
36	Guaiol	1603	1600	2.86
37	2-epi-α-Cedren-3-one	1630	1626	0.24
38	Himachalol	1657	1652	0.31
39	14-hydroxy-9-epi-(E)-Caryophyllene	1672	1668	0.84
40	C10-nor-alamenen-10-one	1700	1702	1.21
	Total			97.24
	Monoterpenes			36.66
	Sesquiterpenes			60.58

RI^a^ = Retention indices calculated from retention times in relation to those of a series C8–C40 of *n*-alkanes on a 30 m DB-5 capillary column. RI^b^ = Retention indices from literature. RI = Retention Indices.

**Table 2:** Total phenolic compounds and antioxidant activity of *C. verbenacea* essential oil.

Analyses	EOCV
ABTS (μmol TE/mL)	915.32 ± 17.27
DPPH (μmol TE/mL)	102.47 ± 6.60
Phenolic compounds (mg GAE/mL)	1.20 ± 0.04

Values represent mean ± SD.

**Table 3:** Minimal Inhibitory Concentration (MIC) and Minimal Bactericidal Concentration (MBC) of *C. verbenacea* essential oil.

Microorganisms	MIC (µL/mL)	MIC *p* Value	MBC (µL/mL)	MBC *p* Value
*B. cereus*	11.3 ^b^	0.0000	20.0 ^d^	0.0001
*S. aureus*	12.5 ^b^	0.0000	25.0 ^c^	0.0000
*E. faecalis*	15.0 ^c^	0.0000	25.0 ^c^	0.0000
*A. baumannii*	25.0 ^a^	0.0001	50.0 ^b^	0.0001
*K. pneumoniae*	25.0 ^a^	0.0001	75.0 ^a^	0.0001
*E. coli*	12.5 ^b^	0.0000	25.0 ^c^	0.0000
*C. albicans*	6.30 ^d^	0.0001	12.5 ^e^	0.0001
*C. krusei*	12.5 ^b^	0.0000	25.0 ^c^	0.0000

Different letters within the same column mean significant differences at *p* < 0.05 according to the Tukey test at the 95% confidence level.

**Table 4:** Characterization of the nanoemulsion of *Cordia verbenacea* essential oil.

Size (nm)	PDI	Zeta Potential (mV)	pH	Spreadability (cm)
126.60 ± 0.83	0.49 ± 0.40	−18.50 ± 0.45	5.30 ± 0.10	4.70 ± 0.15

Values represent mean ± SD.

## Data Availability

Data can be provided by the authors upon reasonable request.
